# The Nasopalatine Ducts Are Required for Proper Pheromone Signaling in Mice

**DOI:** 10.3389/fnins.2020.585323

**Published:** 2020-11-19

**Authors:** Dana Rubi Levy, Yizhak Sofer, Vlad Brumfeld, Noga Zilkha, Tali Kimchi

**Affiliations:** ^1^Department of Neurobiology, Weizmann Institute of Science, Rehovot, Israel; ^2^Department of Structural Biology, Weizmann Institute of Science, Rehovot, Israel

**Keywords:** VNO, nasopalatine ducts, active pumping, nasal and oral cavity, pheromones, social behavior, mice

## Abstract

The vomeronasal organ (VNO) specializes in detection of chemosignals, mainly pheromones, which control social communication and reproduction in many mammals. These pheromones must solubilize with nasal fluids before entering the VNO, and it was suggested that they are delivered to and cleared from the VNO by active pumping. Yet, the details of this pheromone delivery process are unclear. In this study, we first constructed a high-resolution 3D morphological image of the whole adult mouse snout, by using ultra-high-resolution micro-CT. We identified a net of micro tunnels starting from the nostrils and extending around and through the VNO. These micro tunnels connect the nasal cavity with the VNO and the oral cavity via the nasopalatine ducts (NPD). Other micro tunnels connect the nasal cavity to the main olfactory epithelium. We next demonstrated that physical obstruction of the NPD severely impairs the clearance of dissolved compounds from the VNO lumen. Moreover, we found that mice with blocked NPD display alterations in chemosignaling-evoked neuronal activation in brain regions associated with the vomeronasal system. Finally, NPD-blocked male mice exhibit reduced preference for female chemosignals, and impaired social interaction behavior. Taken together, our findings indicate that the NPD in mice are connected to both the nasal and oral cavity, serving an essential role in regulating the flow of soluble chemosignals through the VNO, and are required for proper pheromone-mediated social communication.

## Introduction

The vomeronasal organ (VNO) plays a key role in detecting sex-specific and species-specific chemical signals (Keverne, 1999; Stowers et al., 2002; Dulac and Torello, 2003; Halpern and Martıìnez-Marcos, 2003; Dulac and Kimchi, 2007; Isogai et al., 2011; Beny and Kimchi, 2014; Bear et al., 2016; Marom et al., 2019). In most animal species, VNO-mediated signals regulate a variety of innate behaviors crucial for the survival of the individual and the species (Bielsky and Young, 2004; Brennan and Zufall, 2006; Chamero et al., 2007; Baum and Kelliher, 2009; Haga et al., 2010; Papes et al., 2010; Grinevich and Stoop, 2018). An accumulating body of evidence reported that the mammalian VNO opens into the nasal cavity through a sole opening in its anterior end (Wöhrmann-Repenning, 1984; Mohrhardt et al., 2018). In mice, it is assumed that chemosignals are delivered to the VNO through the nostrils, by active sniffing (Vaccarezza et al., 1981; Dulac and Torello, 2003), while the VNO serves as an active pump to guide molecules into its epithelium (Eccles, 1982; Meredith, 1994). The VNO itself is secluded from airflow and is engulfed by a bony capsule; thus, chemosignals delivered into the nostrils (by sniffing) must solubilize with nasal fluids, before entering the VNO through active pumping (Meredith and O’Connell, 1979). Yet, a characterization of the full process by which chemosignals flow through, and especially cleared from the VNO lumen to allow delivery of new chemosignals, is still missing.

In reptiles, chemosignals reach the VNO only through the mouth – via two fine tubular structures in the anterior part of the palate, termed the nasopalatine ducts (NPD) (Halpern and Martıìnez-Marcos, 2003). In mammals, these ducts create a direct and continuous passage between the nasal and oral cavities, and present high evolutionary conservation across terrestrial vertebrate species (Wohrmann-Repenning, 1980, 1993; Shimp et al., 2003). Felids and ungulates, for example, utilize the NPD for pheromone transfer to the VNO by performing the distinct “flehmen” behavior, in which an animal curls back its upper lip exposing its front teeth and inhales, with the nostrils usually closed (Stahlbaum and Houpt, 1989). Despite the fact that the NPD are clearly present in rodents (Shimp et al., 2003), their role in rodent chemosignaling and in related behaviors has been usually overlooked, and the few studies which explored their functions yielded inconclusive results (Meredith et al., 1980; Mackay-Sim and Rose, 1986; Meredith, 1991). Consequently, there is uncertainty as to whether, and how, the NPD are involved in the process of rodent VNO-mediated chemosignaling.

Here, using high-resolution micro-computerized tomography (micro-CT), together with *in vivo* fluorescent tracing, we explored the flow path of liquid-borne compounds from the nasal cavity to the vomeronasal epithelium and the oral cavity via the NPD. Moreover, by physically obstructing the oral openings of the NPD, we examined their functional role in chemosignaling-evoked neuronal activity and in related social behaviors.

We demonstrate that the rodent NPD are not solely evolutionary remnant anatomical structures, but rather a key element in the biomechanical mechanism that allows proper transportation of compounds from the nose to the sensory luman of the VNO and to the oral cavity. Also, we report the presence of striated muscles around the oral entrances of the NPD. We hypothesize that these muscles can allow voluntary contraction or expansion of the NPD openings (i.e., contraction of the surrounding muscles will open the NPD opening and will allow the influx of fluid from the VNO lumen to oral cavity), and thus enabling a controllable inflow of substances through the sensory epithelium of the VNO. Finally, we report that obstruction of the NPD alters VNO-mediated social behaviors, as well as socially-related neuronal signaling, but apparently does not alter signal processing through the main olfactory system.

## Materials and Methods

### Animals

Mature, sexually naïve, male C57BL/6 mice (Harlan Laboratories, Israel) were used in this study. Mice were maintained on a reverse 12/12 h light/dark cycle, with food and water *ad libitum*. All experimental procedures were approved by the Institutional Animal Care and Use Committee of the Weizmann Institute of Science.

### Micro-CT

Mice were sacrificed and the oral openings of their NPD were filled with radio opaque light curing hybrid composite with flowable viscosity (FLOWline, Heraeus Kulzer, Inc., South Bend, IN, United States), in order to keep the ducts open during fixation and allow clear vision of their location and structure in the micro-CT scan. This composite filling is often used for restoring teeth, and thus appears in our CT scan in white color, same as the teeth. The FLOWline composite is applied as a viscos fluid which then solidifies (thus it did not reach any other spaces or tunnels within the nasal and oral cavities). The upper jaw of the mice was then removed and placed overnight in 4% paraformaldehyde fixative solution (PFA). Following fixation, samples were stained for 48 h in Lugol solution (10 g KI and 5 g I_2_ in 100 ml water) diluted 1:4 in DDW to generate an isotonic medium which minimizes the shrinkage of the soft tissue (Kelly, 1961; Degenhardt et al., 2010). Samples were then immobilized and sealed in a cylindrical holder made of polycarbonate. In order to avoid excessive tissue drying during the measurement, a small piece of wet cloth was placed at the bottom of the holder. This ensured water vapor saturated atmosphere around the sample for the whole duration of the imaging procedure (about 30 h). The holder was then firmly inserted into the sample support of the micro-CT instrument (Micro XCT-400, Xradia, Ltd., Pleasanton, CA, United States). For the micro-CT scan, we set the X-ray source at 40 kV and 200 μA and took projection images with an objective having a nominal magnification of 0.5x. The scan included 6,000 such images taken with 5 s exposure time. No source filter was used. After volume reconstruction (done by the XRadia software which uses the Feldkamp algorithm for filtered back projection), we obtained final 3D images with 10 μm resolution. Further image analysis was performed using Avizo software package (VSG, Ltd., Bordeaux, France).

### Tissue Processing and Histological Analysis of the Mouse Snout

Mice were sacrificed and their upper jaw was placed in 4% PFA for a period of 7-days. Following fixation, the tissue was placed in 10% EDTA solution in room temperature for 10 days to allow decalcification (solution was changed every 3 days). The tissue was then washed in distilled water for 2 h and in 50% ethanol for 30 min, before being embedded in paraffin. Coronal sections (7 μm) of the complete palate and nasal cavity of each mouse were serially cut and mounted onto glass slides. The slices were stained using standard Hematoxylin-Eosin protocol (Burck, 1973), and visualized under a light microscope.

### Nasopalatine Ducts Blocking

Mice were randomly divided into experiment group (*blocked*) and sham operated group (*sham*). Animals were deeply anesthetized using Ketamine (100 mg/kg)/Xylazine (23 mg/kg), placed on their back, and their lower jaw was gently opened. A standard surgical cautery system (Gemini cautery kit, SouthPointe Surgical Supply, Inc., Coral Springs, FL, United States) was used to block the oral entrance to the NPD in the *blocked* group. Specifically, the heated tip of the cautery forceps (0.4 mm in diameter) was placed at the entrance of each duct in the upper palate of the mouse, and cauterization was applied until adhesion of the tissue was visually observed (∼500 ms). In the *sham* group, the cautery forceps were placed on the upper palate just below the entrance to the ducts, and cauterization was applied as in the *blocked* group. Animals were monitored daily following the procedure and allowed 2–3 weeks to recover before the onset of experiments.

#### Confirmation of NPD Obstruction

For visual confirmation, animals were anesthetized as described above. The NPD openings were carefully examined under a binocular microscope (Nikon SMZ 745T) and photographed. For histological confirmation, at the end of experimental procedures, the upper jaw of the mice was processed as described above in the Hematoxylin-Eosin protocol. Coronal sections were examined to confirm closure of the ducts. Mice with two or more consecutive slices where the entrance to the ducts was not fully blocked were excluded from the analysis (*n* = 5).

### Fluorescence Dye Assay

A 10 μM rhodamine B solution (Sigma Laboratories) was freshly made at the beginning of each experiment week, and kept in 4°C, in the dark, for a maximum of 7 days. Experimental mice from both groups (*blocked, sham*) were gently held in place and a total of 3 μl of dye-mixture solution was gradually applied to their left nostril while allowing the mice to freely sniff the solution. Additional control group (*blank*) was comprised of *sham* mice that did not receive any stimulus, and used to quantify baseline auto-fluorescence levels in untreated VNO. Immediately after the dye-stimulus mixture was delivered, mice were euthanized, and their upper jaw was extracted and washed in 0.1M PBS solution. The upper palate was then removed, and the VNO was extracted bilaterally and washed with PBS to remove all traces of nasal mucosa and excessive dye. For measurement of fluorescence intensity, images of both VNOs were taken using a fluorescence stereomicroscope (Leica MZ FL III, Leica, Switzerland). Measurements of fluorescence were assessed using ImagePro Plus software (Media Cybernetics, Rockville, MD, United States). Mean optical density values were separately extracted for each side of each VNO, and then averaged to obtain a single optical density value per mouse.

### Behavioral Assays

#### Olfactory Preference Tests

Mice were individually housed for 1–2 weeks before initiation of behavioral assays. Prior to each experiment, pre-tests were conducted to exclude side preference in the testing apparatuses/home cage. At the beginning of each experiment day, animals were moved to the experiment room and allowed at least 1 h to acclimate. For the odor preference assay, two applicators with cotton tips containing the different stimuli were attached to opposite walls of the home cage (Supplementary Figure S5). On the first day of the experiment, mice were presented with one *“control stimulus”* (saline) and one *“social/non-social odor stimulus”* (200 μl, male/female urine for social odor or banana/cinnamon for non-social odor); on the following day, mice were presented with one “*control stimulus,”* and the complementary *“social/non-social odor stimulus.”* Predator, vaginal secretion and saliva preference assays were conducted using a 3-chamber apparatus as previously described (Karvat and Kimchi, 2013; Beny and Kimchi, 2016; Beny-Shefer et al., 2017; Zilkha et al., 2017). For predator signals, soiled rat bedding was placed in a polycarbonate cup (5 cm height x 7.5 cm diameter). Saliva (100 μl) and vaginal secretion (50 μl) stimuli were presented on microscope slides attached to the chambers’ floor. Sniffing duration for each stimulus were analyzed using the Observer XT and Ethovision XT softwares (Noldus Information Technology, Wageningen, Netherlands Noldus). Mice with total sniffing time of less than 5% of overall experiment duration were excluded from the analysis. Absolute sniffing durations of each stimulus were calculated per mouse by subtracting time spent sniffing the control stimulus [e.g., *female exploration* = *duration sniffing female urine (sec) – duration sniffing saline (sec)*].

For urine stimuli, fresh urine was collected from 8 to 10 adult male or female C57BL/6J mice. Urine stimuli were diluted 1:1 by volume with saline. For control stimulus, standard saline solution was used. For predator stimuli, 30 ml of soiled-bedding was collected from an adult Wistar rats cage, while the same amount of clean bedding was used as control stimulus. Saliva stimuli were collected from 15 adult male and 15 adult female C57BL/6J mice. Mice were anesthetized using Ketamine (100 mg/kg)/Xylazine (23 mg/kg) and exacerbated saliva secretion was induced via *Pilocarpine* injection (0.025%, 100 μl, i.p.). Saliva was diluted 2:3 by volume with saline, and standard saline solution was used as control stimulus. Vaginal secretions were collected from seven adult female mice as previously described (McLean et al., 2012; Scott et al., 2015), with DDW used as control stimulus. For general odors, commercial cinnamon and banana odorant were used (Sensale, Ramat Gan, Israel).

#### Resident Intruder Assay

Two weeks before the initiation of behavioral assays, blocked and sham resident mice were housed in single cages in reversed 12/12-h light/dark cycle. At the beginning of each experiment day, animals were moved to the experiment room and allowed at least 1 h to acclimate. All behavioral procedures were performed during the dark phase under dim red light. Intruder females were C57BL/6J sexually naive 9 weeks old mice. A day prior to the experiment, the females were exposed to soiled male bedding in order to induce an estrous state. Intruder males were C57BL/6J sexually naïve 5 week-old mice. Prior to their introduction into the resident’s home cage, the males were swabbed on their back and anogenital region with 140 μl urine collected from sexually mature and experienced male mice.

Resident male mice were introduced to the intruder in their home cage, and allowed to freely interact for 15 min. Social behavior was observed and recorded using digital video cameras, and analyzed offline using the Observer software (Noldus). The following behavioral parameters were measured: social interaction (sniffing rear and front body parts), sexual behavior (mounting, pelvic thrusts, copulation), aggression (chasing, fighting, rolling, biting) and locomotion activity (digging, grooming, exploration), as previously described (Chalfin et al., 2014; Beny-Shefer et al., 2017).

#### Food Finding Assay

Twenty-four hours prior to the experiment, food was removed from the home cage of the experimental mice and replaced with a small amount of food reward (one pine nut, ∼0.1 g), in order to avoid food neophobia during behavioral tests. Before each trial, a single pine nut was randomly placed at the bottom of a large clean cage (20 cm × 35 cm × 18 cm) covered with 2 cm of bedding. Mice were then individually placed in the cage for 5 min and latency to discover the buried food was measured and compared between groups (*blocked, sham*).

### cFos Measurements

Urine was collected from 8 to 10 adult female mice in all stages of the estrous cycle. For control stimulus, double distilled water (DDW) was used. Animals were divided into three experimental groups: (1) *sham* + *DDW*, sham operated mice that were presented with 200 μl of DDW; (2) *sham* + *urine*, sham operated mice that were presented with 200 μl of female urine; (3) *blocked* + *urine*, mice with surgically blocked NPD that were presented with 200 μl of female urine. The night prior to the experiment, mice were individually placed in an empty cage with clean bedding. On the day of the experiment, stimulus (either urine or DDW) was placed on a small, round (∼5 cm in diameter), transparent and open Petri dish and positioned in the middle of the experimental cage. Mice were allowed to freely explore the stimulus for 15 min.

One and half hours after stimulus presentation, mice were euthanized and perfused with cold 0.1M PBS followed by 4% PFA, as previously described (Scott et al., 2015; Beny-Shefer et al., 2017). Brains were removed and post-fixed in 4% PFA for 48 h. Then, brains were sliced into 30 μm free-floating coronal sections and immunostained for cFos as previously described (Scott et al., 2015). Expression of cFos was assessed bilaterally for five sections per anatomical area. Labeled cell nuclei were counted using the ImagePro Plus software, and their amount was divided by area size in each slice to receive density values of number of cells per mm^2^. Analysis was performed for the anterior medial amygdala (aMeA), posterior medial amygdala (pMeA), anterior piriform cortex (aPir) and posterior piriform cortex (pPir), all as indicated in the mouse brain atlas (Paxinos and Franklin, 2003).

### Statistical Analysis

All statistical analyses, unless stated otherwise, were performed by one-way or two-way ANOVA, followed by Fisher LSD *post hoc* comparisons as follows: olfactory preference test was analyzed using two-way ANOVA with experimental group (*sham*/*blocked*) and stimulus (*male*/*female*) as main factors. Group data in the cFos counts was analyzed using one-way ANOVA (sham + DDW, sham + urine, blocked + urine). For the fluorescence dye assay, latency to find the burried food, and social interactions in the resident intruder assay, groups (blocked, sham) were compared using the Mann-Whitney U test. All statistical analyses were performed using the SPSS software (SPSS, Inc., Chicago, United States), and STATISTICA software (StatSoft, Inc., Tulsa, OK, United States). Results are presented as mean ± SEM, and the appropriate significant results are reported in detail when *p* < 0.05.

## Results

### Micro-Architecture of the NPD Presents a Continuous Route Between the Nasal and Oral Cavities via the VNO

**FIGURE 1:**
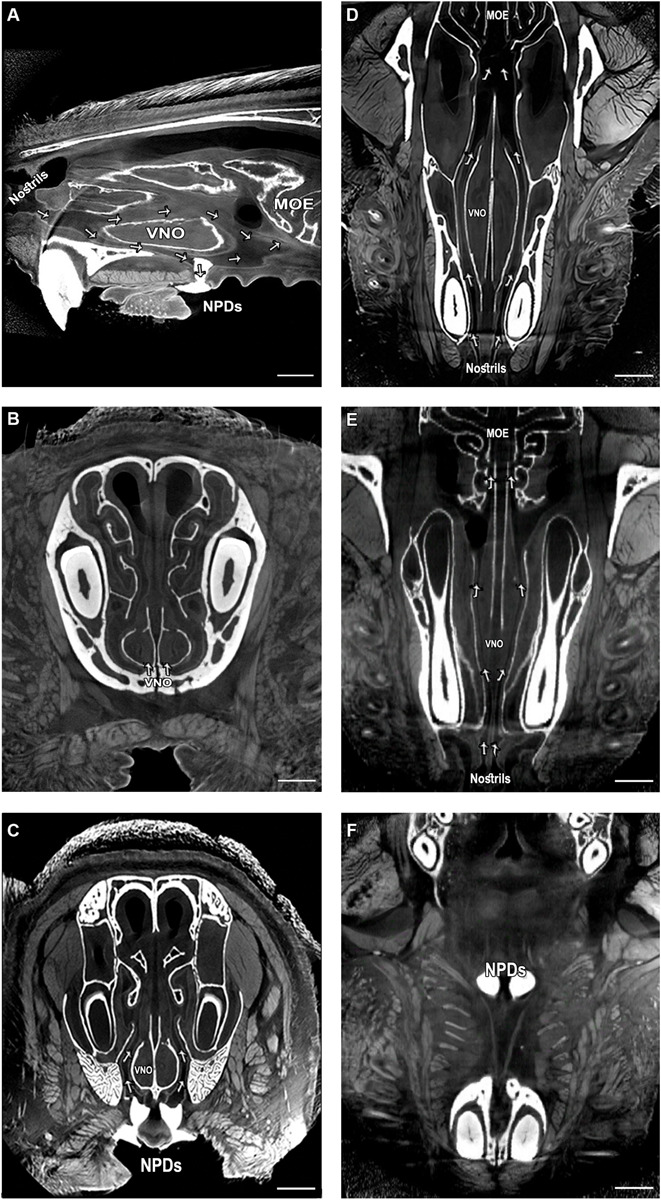
Complex network of micro tunnels connecting the nasal cavity with the VNO and the NPD, which opens to the oral cavity; High-resolution micro computerized tomography (micro-CT) imaging of the mouse snout; **(A)** sagittal, **(B,C)** coronal and **(D–F)** transverse planes of a micro-CT scan (10 μm resolution). The NPD are filled with dental high-contrast substance shown in white. The micro-CT scan revealed a complex network of pathways connecting the nasal cavity with the oral cavity and the VNO (indicated by arrows, see Supplementary Videos S1–S3). The teeth and other bones are seen in white, as are the NPD which were filled with white dental filling. Soft tissues appear in gray, spaces and tunnels appear in black. VNO, vomeronasal organ; MOE, main olfactory epithelium; NPD, nasopalatine ducts. Scale bar: 1 mm.

We first examined whether the NPD maintain an open passageway connecting the oral cavity with the murine VNO. We utilized a high-resolution micro-CT scanning technique with custom-designed methodology in order to reconstruct the complete 3D morphological architecture of the nasal cavity and the NPD of mice (Figure 1 and Supplementary Videos S1–S3). The scans show that much like in reptiles, the murine NPD might constitute a direct passageway connecting the oral cavity and the VNO, in a unilateral way (i.e., the left nostrils leads to the left VNO and then to the left NPD, and same for the right side; see also Supplementary Figure S2). The images show that the NPD (filled with white dental filling) open near the posterior end of the VNO, and continue to the nasal cavity, while creating a clear route between the nasal cavity, the VNO and the mouth (Figure 1 and Supplementary Videos S1–S3). Specifically, the scans revealed a net of micro tunnels (∼100 μm in diameter, marked in arrows) which start at the nostrils and then branch into two main routs: one leads to the rear end of the nasal cavity, while the other leads to the VNO (Figures 1D,E). The tunnels reaching the VNO run between the bone capsule and the VNO lumen, and connect to the NPD, thus creating a continuous nostrils-VNO-mouth track (Figures 1A,C–F). The second passage connects the nasal cavity to the main olfactory epithelium. In addition, we identified both anterior and posterior openings for the VNO capsule (Figure 1E). The posterior opening appears to be directly connected to the network of micro tunnels leading to the NPD, and could potentially enable a passageway for chemosignals between the oral cavity, VNO and nasal cavity. Finally, we conducted a histological examination of the whole mouse snout and noticed the existence of dense striated muscles surroundings the oral entrances of the NPD (Supplementary Figure S1). The position of the muscles suggests that their contraction leads to the opening of the passage connecting the VNO and the oral cavity (i.e., contraction of the muscles would pull away the connective tissue surrounding the opening, thereby opening the ducts). This may indicate a voluntary ability of the mouse to regulate substance flow through the NPD, in case contracting and expanding these muscles indeed modulate opening of the ducts.

### The NPD Are Required for the Proper Clearance of the VNO

**FIGURE 2:**
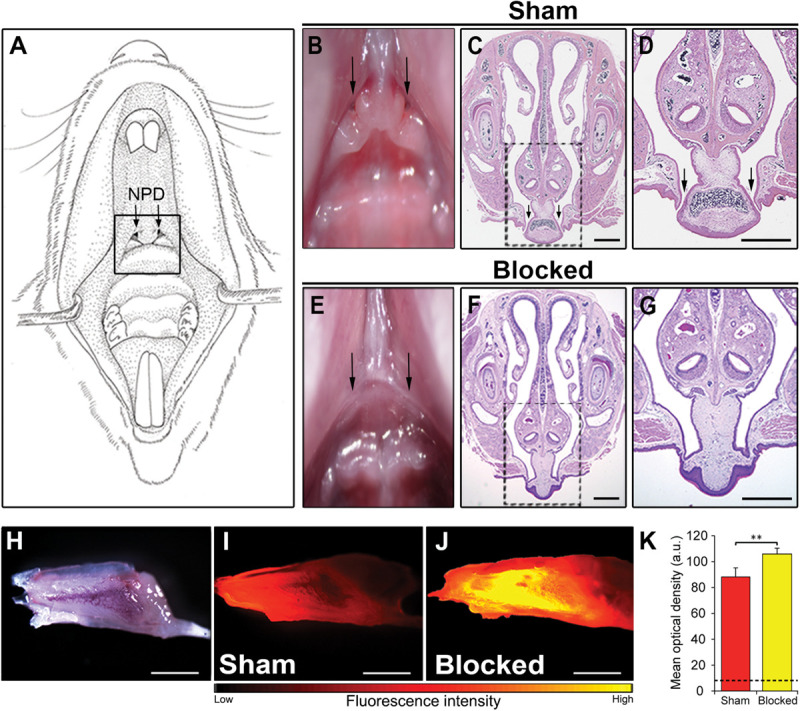
Obstruction of the NPD openings disrupts flow of substances through the VNO. **(A)** Schematic illustration of the oral cavity of an adult mouse. Location of the oral openings of the NPD is indicated by a black rectangle. **(B,E)** Image of the upper palate of a mouse. Arrows in the oral cavity indicate the location of the two openings of the NPD in the sham **(B**) and blocked **(E)** groups. Coronal section through the snout and upper palate of a mouse with intact **(C,D)** or cauterized **(F,G)** NPD, stained with standard hematoxylin-eosin staining. Rectangles in **(C,F)** are enlarged in **(D,G)** respectively. Black arrows indicate the oral openings of the NPD. **(H)** Whole mount untreated VNO as seen under bright illumination. Representative images of the VNO extracted from *sham* **(I)** and *blocked* **(J)** mice, following active nasal inhalation of rhodamine-tagged (red) liquid. Excess accumulating of liquid can be seen in the VNO of the *blocked* group. **(K)** Quantification of fluorescence signals in the VNO following rhodamine treatment in *sham* (red) and *blocked* (yellow) groups. Dashed black line represents baseline mean optical density measured in control *sham* mice that did not receive any rhodamine (*blank*). Scale bar: 1 mm. **p < 0.01. a.u., arbitrary units.

We detected the outer location of the NPD openings in the upper palate of a mouse, on the border between the soft and the hard palate (Figures 2A,B). Considering this location, near the posterior part of the VNO, we speculated that the NPD create a pathway for clearance of substance from the organ. To test this hypothesis we established a novel technique to obstruct the NPD without damaging the VNO or the oral and nasal cavities. To do so, we used a standard cautery unit and applied it to the oral entrances of the NPD found in the upper palate of adult male mice (Figures 2A–D), until adhesion of the local soft tissues was observed (*blocked* group) (Figures 2E–G). As control, we defined a *sham* group, where the cautery forceps were placed on the upper palate just below the openings, and cauterization was applied to the adjacent palate tissue as in the *blocked* group (Figures 2B–D).

To test whether such a procedure will indeed impair the VNO’s flow mechanism, we used a simple yet robust paradigm described in a study by Wysocki et al. (1980). We first allowed *blocked* and *sham* adult mice to sniff a rhodamine-stained solution. About 1 min after a rhodamine drop positioned on their nostril was sniffed into the nasal cavity, the mice were sacrificed. We then measured the subsequent fluorescence levels in the VNO as an indication of the amount of substance to reach the lumen. In line with our hypothesis, the results show that all *blocked* mice presented an abnormal accumulation of dyed solution in their VNO (Figures 2H–K and Supplementary Figure S2). Quantification of this signal revealed significantly higher levels of fluorescence in the VNO of *blocked* mice when compared to *sham* mice (*n*_sham_ = 7, *n*_blocked_ = 7, *z* = 2.747, *p* = 0.004; Figure 2K). This indicates that substances reaching the VNO are not properly cleared out in *blocked* mice, possibly impairing the pumping mechanism of the VNO.

### Obstructing the NPD Impairs Chemosignaling-Evoked Neuronal Activation

**FIGURE 3:**
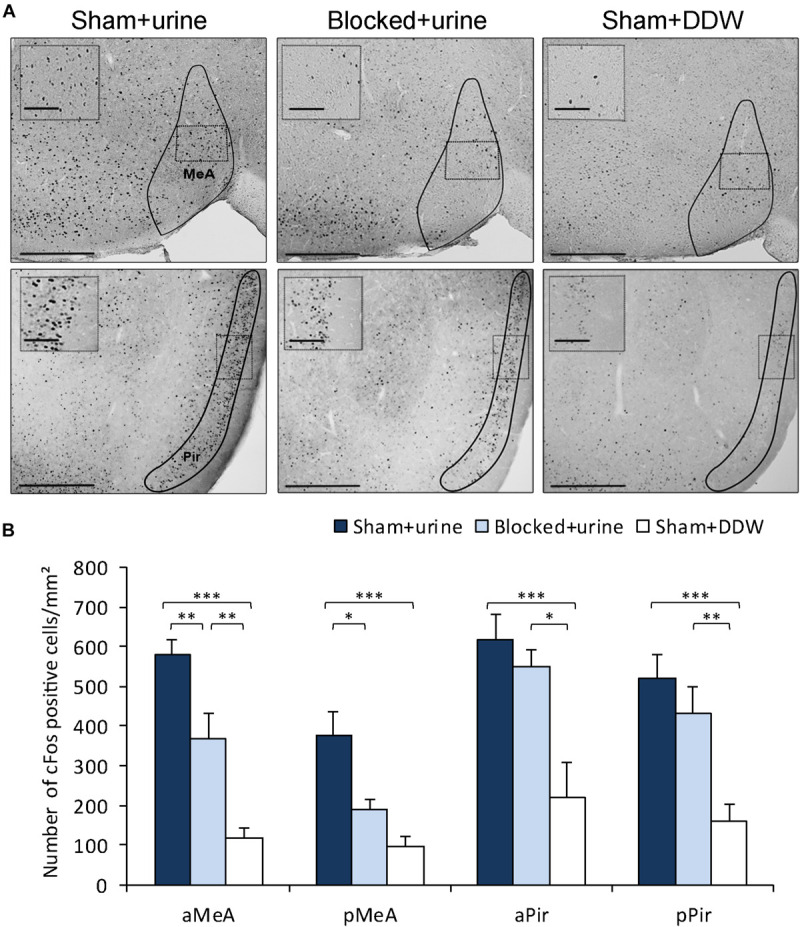
Obstruction of the NPD reduces pheromone-induced neuronal activity in the MeA. **(A)** Representative images of cFos staining in coronal sections of *sham* + *urine* (left panels), *blocked* + *urine* (middle panels), and *sham* + *DDW* (right panels) mice. Anatomical areas of interest are outlined in black. Insets depict areas outlined by dotted rectangles. **(B)** Quantification of cFos reactivity in secondary chemosignaling processing brain regions. *Blocked* male mice exposed to female urine presented decreased neuronal activity when compared to *sham* mice in the medial amygdala, but not in the piriform cortex. aMeA, anterior medial amygdala; pMeA, posterior medial amygdala; aPir, anterior piriform cortex; pPir, posterior piriform cortex. Scale bar: 500 μm, inset: 100 μm. *p < 0.05, **p < 0.01, ***p < 0.001.

We then tested whether obstruction of the NPD can lead to deficits in VNO-mediated detection of chemosignals. Such impairment could be manifested in altered pheromone-evoked neuronal activity in brain regions involved in social chemosignals processing. To test this possibility, we first exposed male mice from both experiment groups (*blocked* and *sham*) to female urine (*blocked* + *urine* and *sham* + *urine*, respectively). An additional control group comprised of *sham* mice that were exposed only to distilled water (*sham* + *DDW*), as a measurement of cFos baseline activity. We measured neuronal activity levels in these groups by quantifying cFos immunoreactivity in the medial amygdala – a region known to be highly involved in the processing of chemosignals (Meredith and Westberry, 2004; Samuelsen and Meredith, 2009a; Petrulis, 2013; Bergan et al., 2014) and execution of social behaviors (Felix-Ortiz and Tye, 2014; Shemesh et al., 2016). We found that mice with *blocked* NPD present significantly decreased neuronal activity levels in the anterior and posterior MeA when compared to *sham* mice, following active investigation of female urine. Importantly, this reduction was not observed in the piriform cortex, which was used as a control region, related to the main olfactory system (aMeA, *n* = 23, *F*_(__2_,_20__)_ = 30.323, *p* < 0.001; *post hoc p* < 0.01 for *sham* + *urine* vs. *blocked* + *urine*; pMeA, *n* = 24, *F*_(__2_,_21__)_ = 11.652, *p* < 0.001; *post hoc p* < 0.05 for *sham* + *urine* vs. *blocked* + *urine*; aPir, *n* = 25, *F*_(__2_,_22__)_ = 9.724, *p* < 0.001; *p* = 0.9 for *sham* + *urine* vs. *blocked* + *urine*; pPir, *F*_(__2_,_22__)_ = 11.9942, *p* < 0.001; *p* = 0.88 for *sham* + *urine* vs. *blocked* + *urine*; Figure 3).

### The NPD Are Crucial for VNO-Mediated Social Behaviors in Male Mice

**FIGURE 4:**
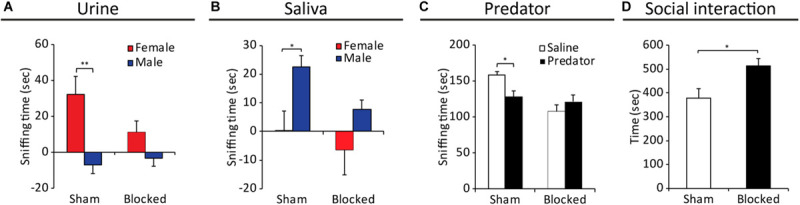
Obstruction of the NPD induces alterations in VNO-mediated behavioral responses of males toward female signals. Mean duration of olfactory investigation in *sham* and *blocked* male mice presented with: **(A)** male/female conspecific urine, **(B)** male/female saliva, **(C)** predator bedding. Blocked mice presented significantly impaired preference for exploration of various chemosignals. **(D)** Significant increase in social investigation of an intruder female, in male mice with obstructed NPD (*blocked*) vs. controls (*sham*). *p < 0.05, **p < 0.01.

Direct impairments in VNO function were repeatedly shown to induce alterations in various conspecific social behaviors (Leypold et al., 2002; Dulac and Torello, 2003; Chamero et al., 2007; Kimchi et al., 2007; Ben-Shaul et al., 2010). Thus, we first examined the effects of blocking the NPD on the innate preference of male mice for female chemosignals (Bean et al., 1986; Beny and Kimchi, 2016; Beny-Shefer et al., 2017). We exposed both *blocked* and *sham* male mice to either saline, female urine or male urine stimuli, which were presented on opposite sides of their home cage (Supplementary Figure S5). We then tested the preference of each mouse by quantifying the duration it spent sniffing each stimulus. The results reveal that *sham* mice presented robust preference for female urine over male urine, while *blocked* mice exhibited no such preference (*n*_blocked_ = 7, *n*_sham_ = 9. *F*_stimulus(__1_,_14__)_ = 12.251; *p* > 0.01; *sham* group: *p* < 0.01 for sniffing female urine vs. sniffing male urine; *blocked* group: *p* = 0.23 for the same comparison, Figure 4A).

As chemosignals are found not only in urine, but also in other body secretions such as saliva (Loebel et al., 2000; Gröschl, 2009; Liberles, 2014) and vaginal secretions (Bell et al., 2013; Liberles, 2014), we further tested for alterations in evoked behavioral responses to these stimuli in control and NPD manipulated mice. We found that while *sham* mice preferred to explore saliva extracted from males over female saliva, *blocked* mice did not exhibit any sex-specific preference (*n*_blocked_ = 12, *n*_sham_ = 9. *F*_stimulus(__1_,_19__)_ = 8.213; *p* < 0.01; *sham* group: *p* < 0.05 for exploring male saliva vs. exploring female saliva; *blocked* group: *p* = 0.11 for the same comparison, Figure 4B). A similar effect was observed for exploration of vaginal secretion as *sham* mice showed a preference trend for exploring social chemosignals over a neutral stimulus (i.e., saline), while blocked mice did not show any clear preference. (*n*_blocked_ = 12, *n*_sham_ = 10, *F*_stimulus(__1_,_20__)_ = 4.652, *p* < 0.05; *sham* group: *p* < 0.08 for exploring vaginal secretions vs. saline; *blocked* group: *p* = 0.253 for the same comparison).

The VNO detects not only conspecific signals, but also danger interspecific signals such as molecules emitted from predators (known as kairomones) (Papes et al., 2010; Liberles, 2014). We found that while control mice tend to avoid predator signals (rat soiled-bedding, in comparison to clean bedding), *blocked* mice did not behaviorally distinguish between the two stimuli, thus lacking the innate chemosignals-mediated predator avoidance response (*n*_blocked_ = 9, *n*_sham_ = 8. For exploration duration: *F*_stimulus__X group__(__1_,_15__)_ = 5.303; *p* < 0.05; *sham* group: *p* < 0.05 for sniffing predator bedding vs. clean bedding; *blocked* group: *p* = 0.317 for the same comparison; Figure 4C).

Finally, to examine the effect of NPD obstruction on social interactions, we introduced mice from the different groups to both male and female intruders, and tested the duration of subsequent social interaction, sexual and aggressive behavior. We found that NPD-*blocked* mice spend significantly more time investigating (i.e., social interaction) a female intruder compared to *sham* mice (*n*_sham_ = 8, *n*_blocked_ = 12, *z* = 2.469, *p* < 0.05, Figure 4D). In addition, the NPD-*blocked* mice exhibited significantly lower locomotion activity in the presence of a female intruder (i.e., cage exploration, *n*_sham_ = 8, *n*_blocked_ = 12, *z* = −3.41, *p* < 0.01). No differences were found in the duration of sexual behavior (*n*_sham_ = 8, *n*_blocked_ = 12, *z* = 0.99, *p* = 0.316). In contrast, no differences in any parameter of social behavior toward male intruders were found between the mice with obstructed NPD and control sham-treated (Supplementary Figure S3).

We also conducted an additional set of behavioral assays designed to test for possible behavioral alterations related to the main-olfactory system (Wilson et al., 2006; Vinograd et al., 2017; Fleming et al., 2018). First, we tested for changes in olfactory preference to non-social odors (cinnamon/banana), and found no differences in preference for these odors between the control and experimental groups (*n*_blocked_ = 9, *n*_sham_ = 9. For duration: *F*_group__(__1_,_16__)_ = 0.014; *p* = 0.99; banana: *p* = 0.8 for *sham* vs. *blocked*; cinnamon: *p* = 0.81 for the same comparison; Supplementary Figure S4A,B). Next, we conducted a buried food-finding assay (Le Pichon et al., 2009), where mice are placed in a cage with a hidden pine-nut that offers only olfactory cues for its location. No differences were found between groups in their latency to retrieve the concealed nut (*n*_sham_ = 14, *n*_blocked_ = 9, *z* = 0.535, *p* = 0.59; Supplementary Figure S4C).

## Discussion

The NPD are widely accepted as an integral part of the chemosignaling system in reptiles, creating the main route for pheromone transfer to the VNO from the oral cavity. In rodents, chemosignals are reported to reach the VNO through the nose by active sniffing (Wohrmann-Repenning, 1980, 1993; Shimp et al., 2003), and the NPD are largely overlooked. The question arises as to which role, if any, do the NPD play in the murine chemosignaling system. Our findings describe, for the first time, a functional role for the NPD in facilitating substance flow through the mouse VNO, enabling the proper pumping mechanism of the VNO. Based on high-resolution CT scans we report that the NPD create a continuous route for delivery of fluid substances from the nostrils to the VNO lumen and the oral cavity. Using *in vivo* fluorescence tracing we have demonstrated abnormal accumulation of fluids in the VNO following obstruction of the ducts, indicating that the mammalian NPD constitute a route for substance clearing from the VNO, and that the flow of substances from the nostril through the VNO and NPD to the oral cavity is unilateral (i.e. each nostril leads to the VNO and NPD on the same side, without crossing to the opposite side, see Supplementary Figure S2). We also found that the oral entrances of the NPD are surrounded by dense striated muscles. Such muscles may be voluntary contracted or expanded to control the openings of the NPD, thus possibly allowing the mouse to regulate the inflow of substances to and from the VNO. Further research is needed to determine whether indeed these muscles regulate the opening and closure of the NPD.

The medial amygdala plays a crucial role in processing of social and predator signals detected by the vomeronasal system (Beny and Kimchi, 2014; Bergan et al., 2014; Chen and Hong, 2018; Mohrhardt et al., 2018). Here, we first show a significant reduction of neuronal response to social stimuli (measured by cFos immunoreactivity) in the medial amygdala following NPD obstruction, consistent with previous studies showing reductions in cFos expression following surgical or genetic ablation of the VNO (Kondo et al., 2003; Hasen and Gammie, 2009; Samuelsen and Meredith, 2009b). The reduced neuronal activity indicates that NPD obstruction significantly impairs the function of the VNO.

We also show that obstruction of the NPD alone, with no perturbations of the VNO or the nasal pathway, resulted in prominent deficits in chemosignaling-evoked behavioral phenotypes, and apparently without inflicting detection of odors via the main olfactory system. Specifically, NPD-obstructed male mice lacked the innate preference toward female pheromones, a deficit that was observed in mice with surgically or genetically ablated VNO (Nyby et al., 1985; Bean et al., 1986; Leypold et al., 2002; Stowers et al., 2002; Pankevich et al., 2004; Kimchi et al., 2007; Martinez-Garcia et al., 2009; Stowers and Logan, 2010). Mice with ablated VNO also present an abnormally increased olfactory investigation behavior, a phenotype we have demonstrated as well in NPD obstructed mice. Moreover, we find that NPD-obstructed mice present impairments in sexual discrimination tasks, in which intact mice display a clear preference toward opposite sex stimuli (Byatt and Nyby, 1986; Bímová et al., 2009; Gröschl, 2009), and show no predator avoidance behavior, typically shown robustly by rodents (Blanchard et al., 1998), indicating deficits in pheromone processing. However, NPD-obstructed mice did not show any deficits in sexual behavior toward females, or in aggressive behavior toward males, unlike genetically or surgically ablated VNO mice (Leypold et al., 2002; Stowers et al., 2002; Dulac and Kimchi, 2007). Interestingly, in female hamsters, both surgical ablation of the VNO or blockage of the NPD impairs sexual behavior (i.e., lordosis) (Mackay-Sim and Rose, 1986). Further research is needed to clarify the mechanistic difference between complete VNO ablation and the partial impairment of VNO functioning caused by NPD blockage.

It was suggested that chemosignaling molecules enter the VNO lumen via an active pumping mechanism (Meredith et al., 1980; Wysocki et al., 1980; Eccles, 1982; Meredith, 1994). The sequestered position of the receptor epithelium within the VNO raised questions concerning the access of chemosignals to the receptors. The seminal work of Meredith and O’cconell has demonstrated a pumping mechanism that is powered by vasomotor movements, which can suck solubilized stimulus substances from the nasal cavity into the VNO lumen (Meredith and O’Connell, 1979). The repetitive pumping action requires both the active insertion and the active expulsion of chemosignals to/from the VNO (Ben-Shaul et al., 2010). Our results further support this notion and suggest that the murine NPD might serve as an essential component in this pump as they facilitate substance outflow from the VNO. This evacuation of fluids is crucial in order to enable the entrance of additional chemosignals into the VNO and the repetitive motion of the pump. Blocking the NPD obstructs the continuous renewal of VNO stimulus, leading to impairments in VNO functioning, without any apparent impairments in main olfactory functions. The impairment in the VNO function leads to deficits in pheromone processing and associated responses, as demonstrated by our behavioral and neuronal analysis. These results resemble part of the physiological and behavioral impairments observed in VNO ablated mice (Leypold et al., 2002; Stowers et al., 2002; Kimchi et al., 2007; Hasen and Gammie, 2009; Beny and Kimchi, 2016).

Considering all the above findings, we suggest that the VNO operates as the biological equivalent of a “valveless pump,” which is also present in other biological systems such as the embryonic heart (Männer et al., 2010). Our data indicates that pheromones travel from the nose, to the VNO, and are expulsed through the NPD, in a possibly controllable manner. This requires the VNO to perform a repetitive pumping motion while the muscles around the NPD contract to expand their openings, and allow substance flow. This evacuation of fluids is crucial in order to allow the entrance of novel pheromones into the VNO, and the repetitive motion of the pump. Thus, blocking the NPD severely obstructs this continuous flow, leading the mechanism to malfunction.

Our findings indicate that the NPD are required for continuous VNO clearance and thus are required for proper pumping of compounds through the VNO, conserving a key functional role in murine chemosignaling.

## Data Availability Statement

All datasets presented in this study are included in the article/Supplementary Material.

## Ethics Statement

The animal study was reviewed and approved by Weizmann Institute IACUC.

## Author Contributions

TK conceptualized the research design. DL and YS performed the fluorescence and behavioral experiments. VB performed the Micro-CT scans and assisted with the analysis of the imaging data. DL, YS, NZ, and TK analyzed the results and wrote the manuscript. All authors contributed to the article and approved the submitted version.

## Conflict of Interest

The authors declare that the research was conducted in the absence of any commercial or financial relationships that could be construed as a potential conflict of interest.

## Supplementary Material

Supplementary Video 1 Micro-CT 3D-reconstruction of the mouse snout: Coronal plane (from anterior to posterior).

Supplementary Video 2 Micro-CT 3D-reconstruction of the mouse snout: Transverse plane (from ventral to dorsal).

Supplementary Video 3 Micro-CT 3D-reconstruction of the mouse snout: Sagittal plane (from medial to lateral).
