# Supplementary figures and images for: The Nasopalatine Ducts Are Required for Proper Pheromone Signaling in Mice
Source: Front Neurosci. 2020 Nov 19;14:585323. doi: 10.3389/fnins.2020.585323 (PMC7710809; doi:10.3389/fnins.2020.585323)

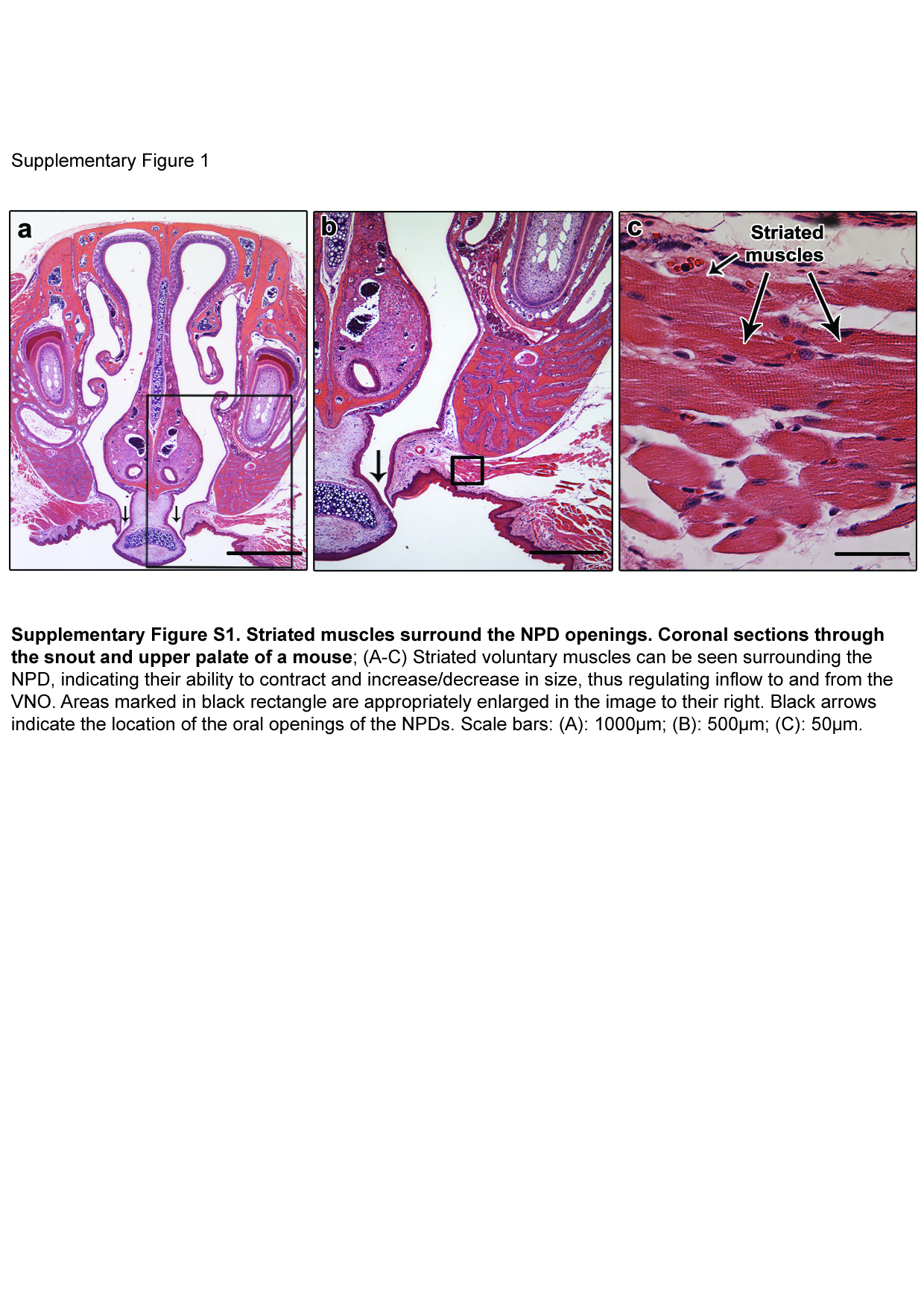

Supplement: Supplementary file 4 [file Image_1.tif]

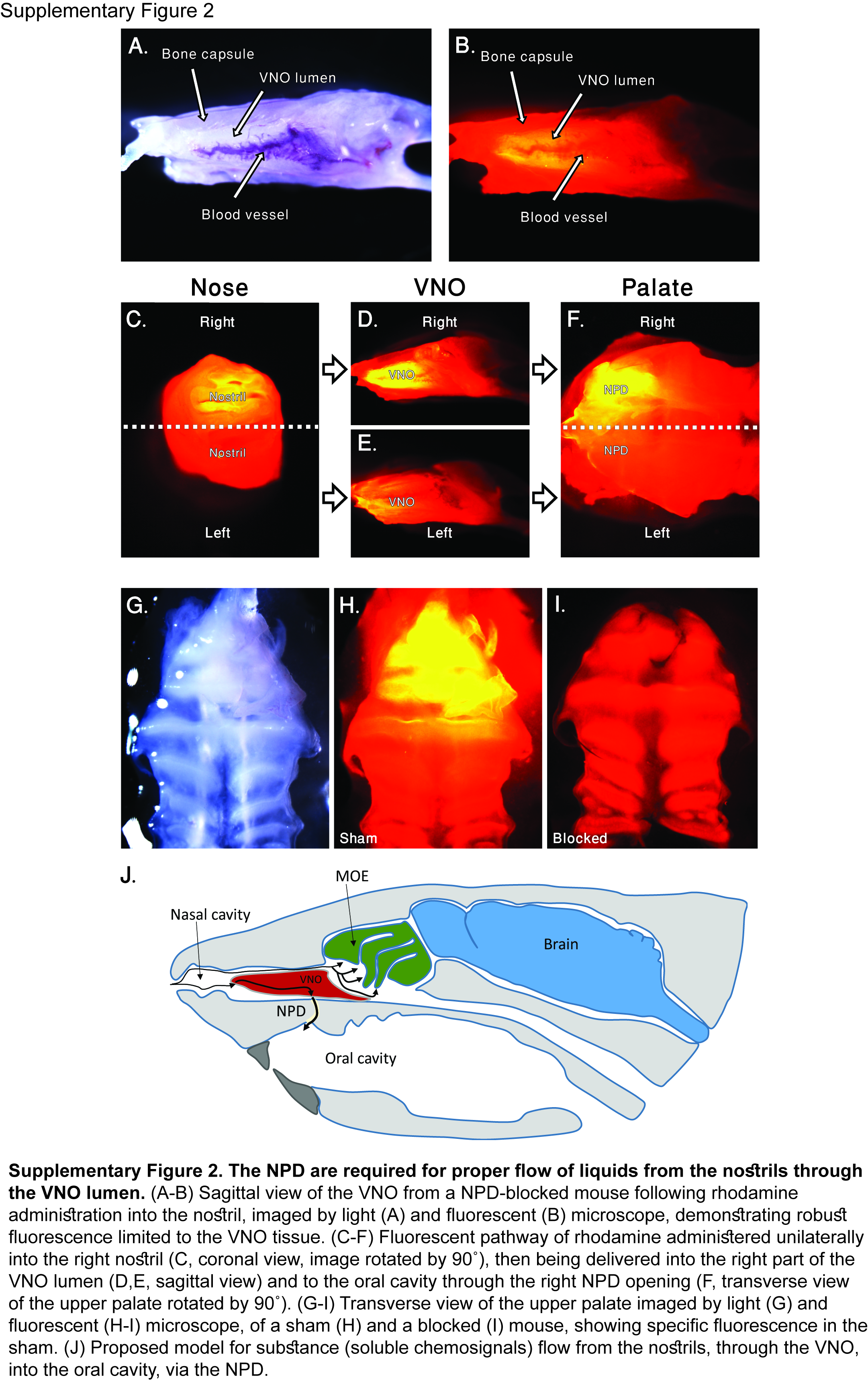

Supplement: Supplementary file 5 [file Image_2.tif]

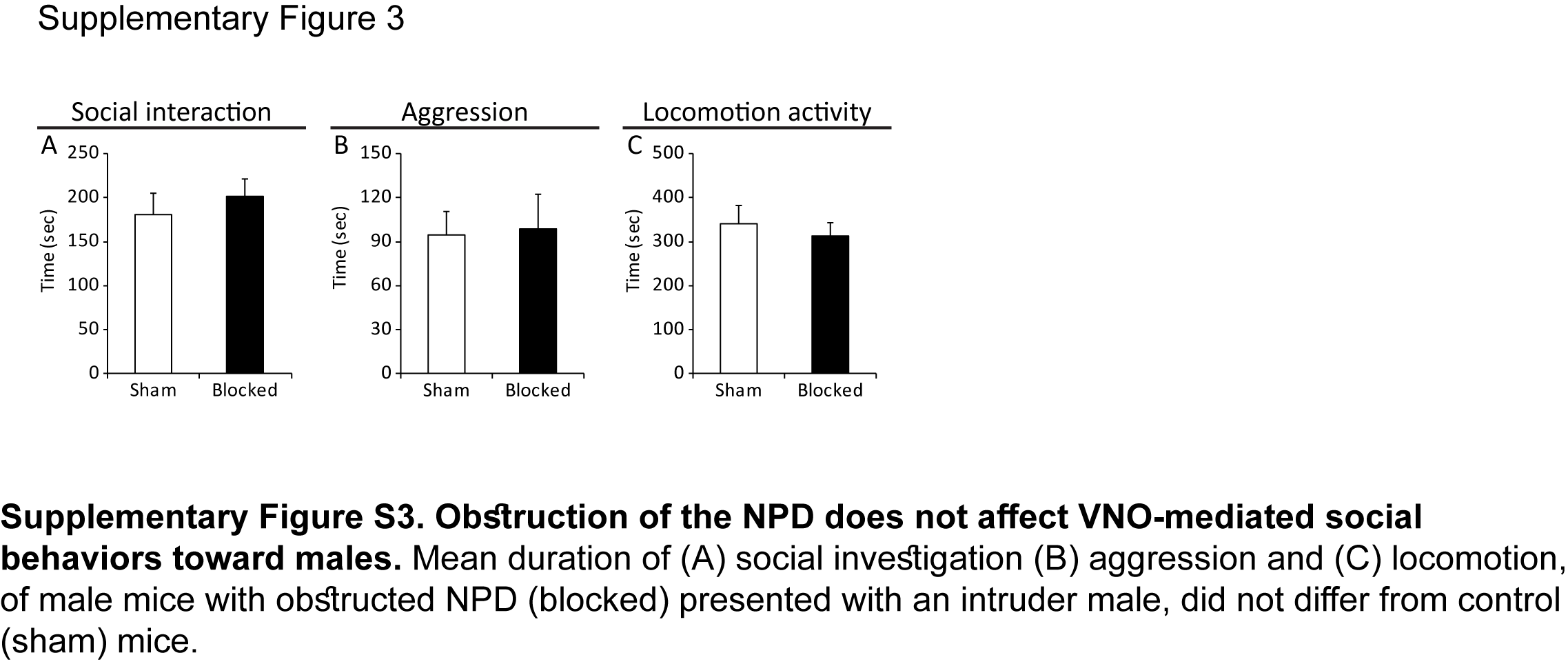

Supplement: Supplementary file 6 [file Image_3.tif]

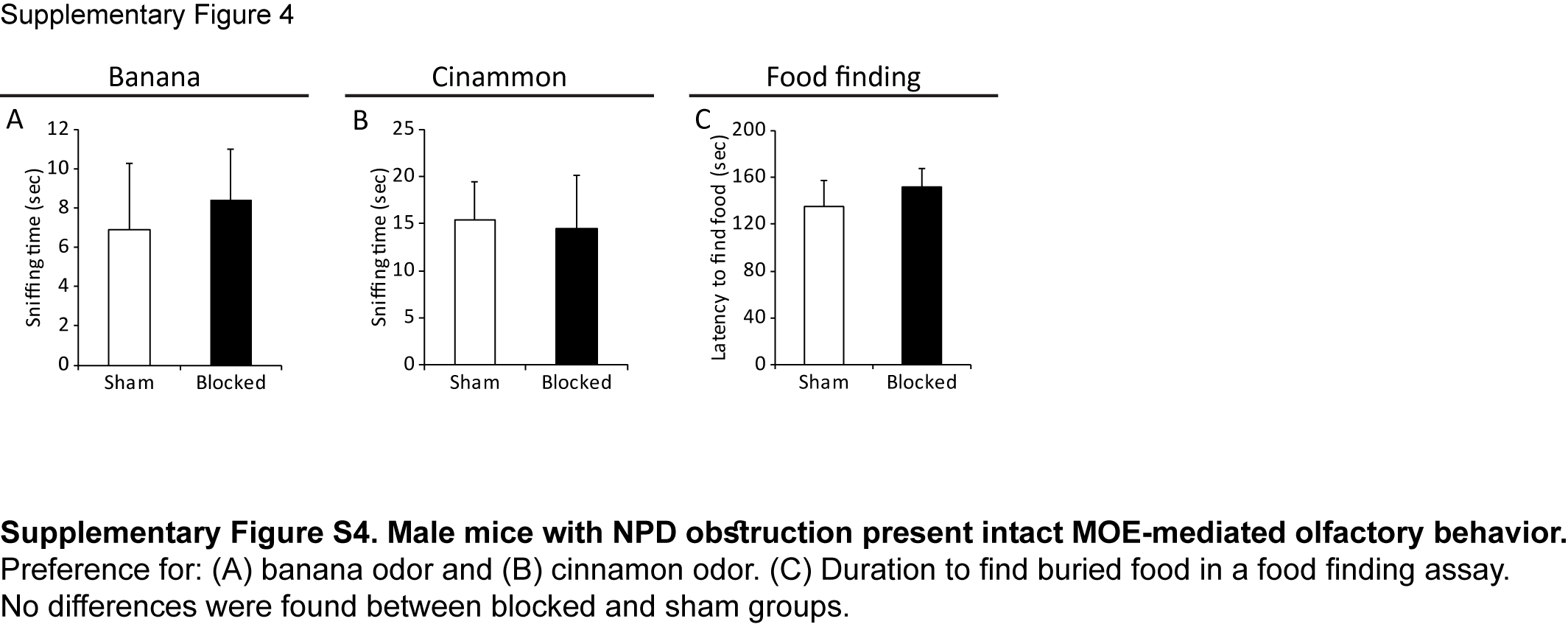

Supplement: Supplementary file 7 [file Image_4.tif]

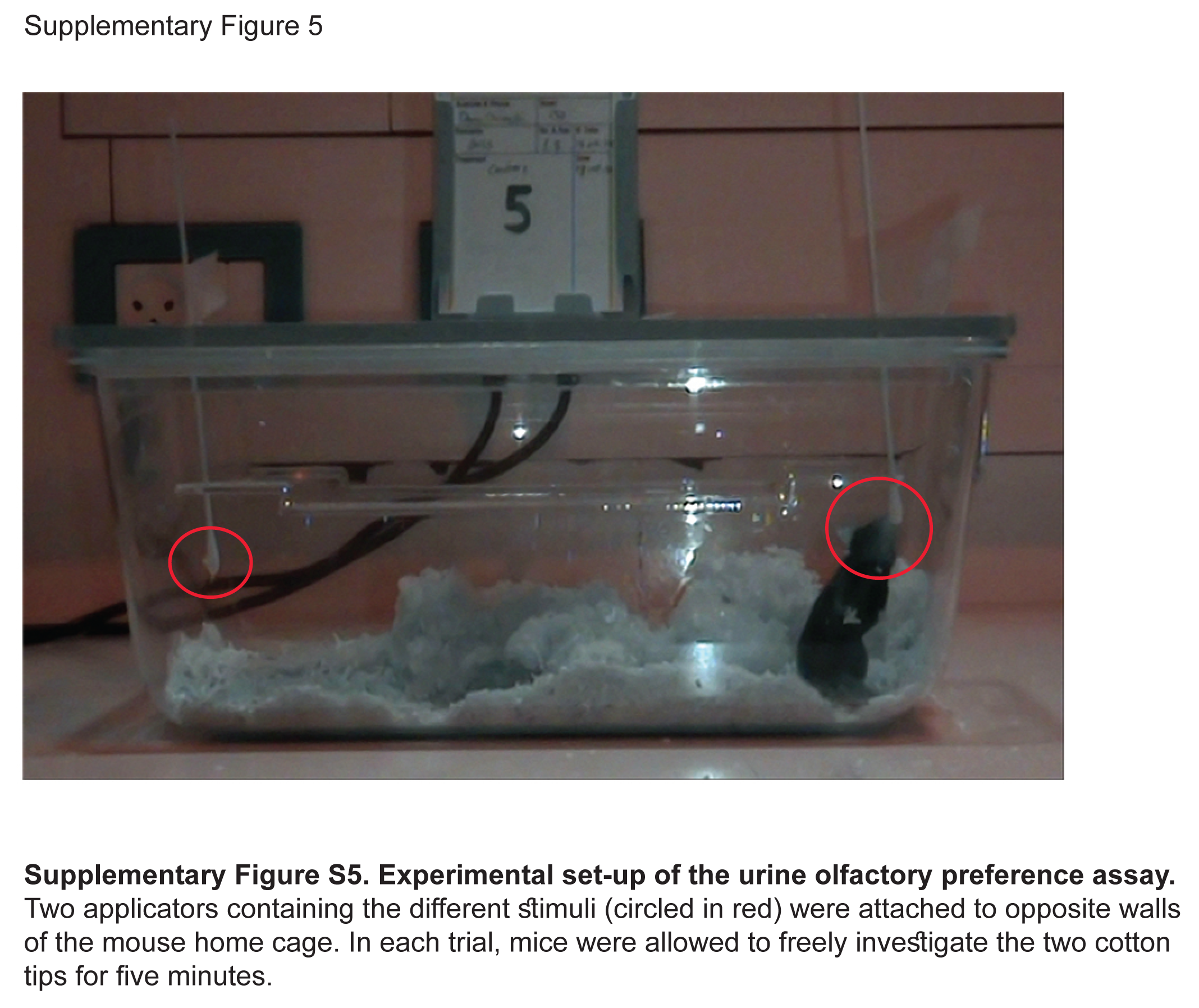

Supplement: Supplementary file 8 [file Image_5.tif]
